# *Vp*StyA1/*Vp*StyA2B of *Variovorax paradoxus* EPS: An Aryl Alkyl Sulfoxidase Rather than a Styrene Epoxidizing Monooxygenase

**DOI:** 10.3390/molecules23040809

**Published:** 2018-04-02

**Authors:** Dirk Tischler, Ringo Schwabe, Lucas Siegel, Kristin Joffroy, Stefan R. Kaschabek, Anika Scholtissek, Thomas Heine

**Affiliations:** 1 Institute of Biosciences, Environmental Microbiology, TU Bergakademie Freiberg, Leipziger Str. 29, 09599 Freiberg, Germany; ringoschwabe007@gmail.com (R.S.); lucasbenedikt@aol.com (L.S.); kristin.friebel@student.tu-freiberg.de (K.J.); stefan.kaschabek@ioez.tu-freiberg.de (S.R.K.); anika.scholtissek@gmail.com (A.S.); heinet@tu-freiberg.de (T.H.); 2 Microbial Biotechnology, Ruhr University Bochum, Universitätsstr. 150, 44780 Bochum, Germany

**Keywords:** sulfoxidation, epoxidation, two-component monooxygenase, flavoprotein, enantioselective biotransformation, fusion protein, protein linker, soil microorganism

## Abstract

Herein we describe the first representative of an E2-type two-component styrene monooxygenase of proteobacteria. It comprises a single epoxidase protein (*Vp*StyA1) and a two domain protein (*Vp*StyA2B) harboring an epoxidase (A2) and a FAD-reductase (B) domain. It was annotated as *Vp*StyA1/*Vp*StyA2B of *Variovorax paradoxus* EPS. *Vp*StyA2B serves mainly as NADH:FAD-oxidoreductase. A *K*_m_ of 33.6 ± 4.0 µM for FAD and a *k*_cat_ of 22.3 ± 1.1 s^−1^ were determined and resulted in a catalytic efficiency (*k*_cat_ *K*_m_^−1^) of 0.64 s^−1^ μM^−1^. To investigate its NADH:FAD-oxidoreductase function the linker between A2- and B-domain (AREAV) was mutated. One mutant (AAAAA) showed 18.7-fold higher affinity for FAD (*k*_cat_ *K*_m_^−1^ of 5.21 s^−1^ μM^−1^) while keeping wildtype NADH-affinity and -oxidation activity. Both components, *Vp*StyA2B and *Vp*StyA1, showed monooxygenase activity on styrene of 0.14 U mg^−1^ and 0.46 U mg^−1^, as well as on benzyl methyl sulfide of 1.62 U mg^−1^ and 3.11 U mg^−1^, respectively. The high sulfoxidase activity was the reason to test several thioanisole-like substrates in biotransformations. *Vp*StyA1 showed high substrate conversions (up to 95% in 2 h) and produced dominantly (*S*)-enantiomeric sulfoxides of all tested substrates. The AAAAA-mutant showed a 1.6-fold increased monooxygenase activity. In comparison, the GQWCSQY-mutant did neither show monooxygenase nor efficient FAD-reductase activity. Hence, the linker between the two domains of *Vp*StyA2B has effects on the reductase as well as on the monooxygenase performance. Overall, this monooxygenase represents a promising candidate for biocatalyst development and studying natural fusion proteins.

## 1. Introduction

Flavin-dependent monooxygenases are able to catalyze a number of biotechnologically important reactions which are often regio- and enantioselective [1,2,3]. These enzymes can be divided into eight groups according to their structure and function [1]. Regio- and enantioselective epoxidation and sulfoxidation reactions are attractive for many purposes [4,5,6,7,8,9,10,11]. Flavin-dependent styrene monooxygenases (SMOs) represent a group performing both reactions with a certain selectivity [2,5,6,7,8,9,10]. This is group E among the flavin-dependent monooxygenases which can be described as follows (EC 1.14.14.11) [1]. These are two-component systems. A strictly NADH-dependent oxidoreductase (StyB; EC 1.5.1.36) produces reduced FAD for the monooxygenase component (StyA, StyA1). Some of these reductases occur as self-sufficient natural fusion proteins (or two domain proteins; StyA2B) composed of an oxygenase (A2) and reductase (B) domain. And those represent the prototype of E2-type SMOs which were first described from a *Rhodococcus* [5]. Most of the monooxygenases (StyA, StyA1) described utilize styrene as major substrate and even belong to natural styrene degradation pathways [11,12]. Recently, some E2-type SMOs have been discussed as initial step of indole detoxification or degradation [13,14]. Thus they can also be described as indole monooxygenases (IMOs).

E-type monooxygenases rely on the flavin cofactor flavin adenine dinucleotide (FAD). The reduced flavin is transferred by diffusion or by direct transfer between both components [15,16,17]. The monooxygenase binds tightly the reduced cofactor and the oxygen driven catalysis gets initiated [15,16,17,18,19]. Here oxygen is activated by the reduced FAD to a (hydro)peroxy-FAD which can attack the actual substrate, e.g., styrene. Upon substrate oxygenation a hydroxyl-FAD intermediate is formed and decomposes to oxidized FAD and water [18,19,20]. The product is subsequently released and another catalytic cycle can start.

E2-type monooxygenases have been shown to be excellent in sulfoxidation with respect to substrate conversion and enantioselectivity whereas the E1-type only shows a high activity at low enantioselectivity [1,2,3,4,5,6,7,8,9,10]. However, so far there is only a single E2-type SMO in detail described and it has a rather low activity [5,6,9]. Therefore, it is reasonable to screen for additional E2-type monooxygenases which can be applied in biocatalysis.

**Scheme 1:**
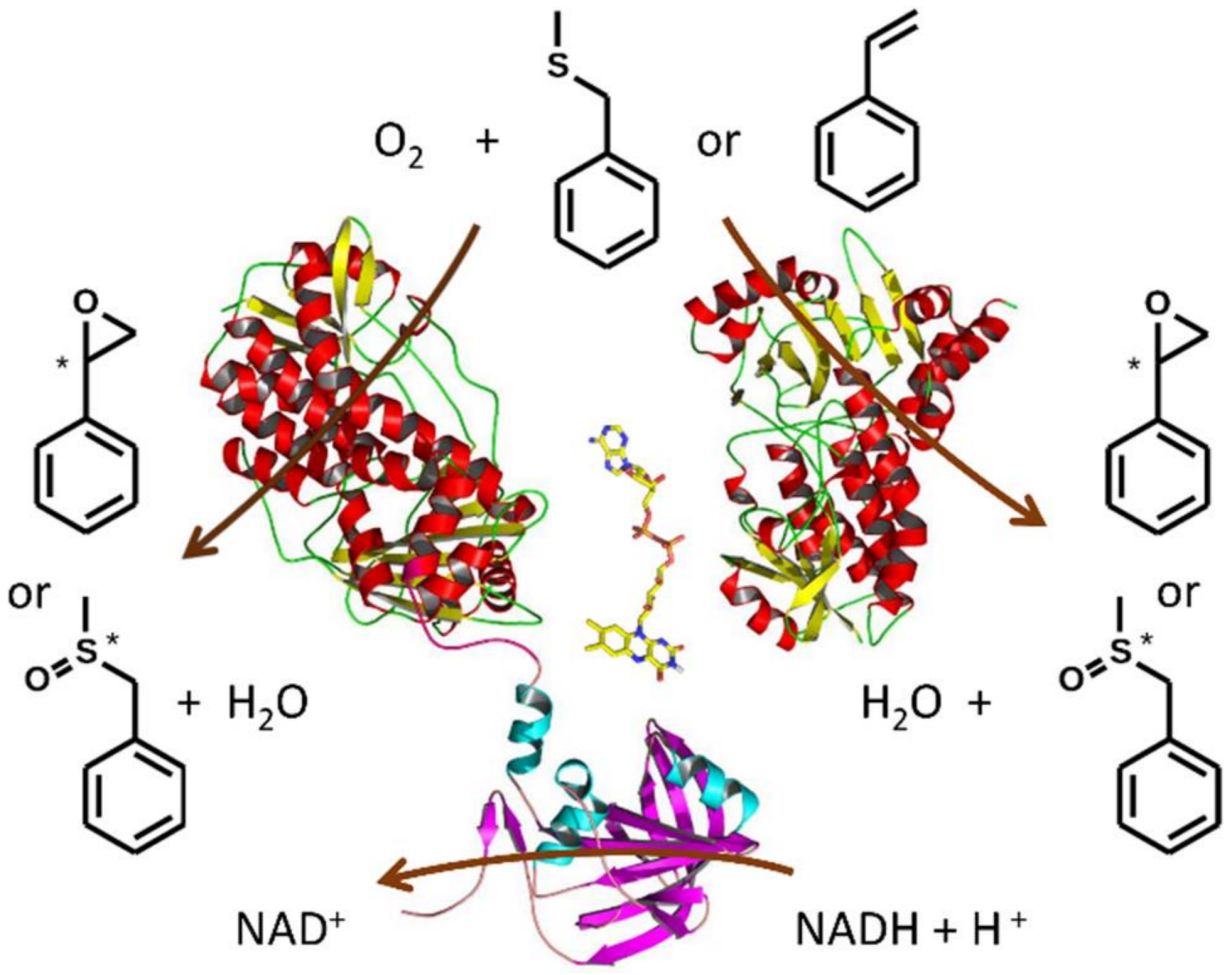
A view on styrene monooxygenase activity. The reductase domain of StyA2B reduces FAD (displayed in oxidized form between protein monomers) upon NADH-consumption. Reduced FAD can be used by both monooxygenase units, StyA1 (major; right) and StyA2 (minor; left), to activate molecular oxygen and to subsequently monooxygenate the substrates (here for example styrene and benzyl methyl sulfide) [5,6,7,8,9,10]. Upon substrate oxygenation hydroxyl-FAD is formed and thereof water is eliminated to recycle the FAD in its oxidized form for the next catalytic cycle.

Based on former studies of E2-type SMOs [4,5,6,9,11,21,22] it was reasonable to investigate the phylogenetic more different system *Vp*StyA1/*Vp*StyA2B of *Variovorax paradoxus* EPS (accession numbers: ADU39063 and ADU39062). The general activity and capability to convert styrene but also sulfides in comparison to other monooxygenases is presented (Scheme 1).

## 2. Results and Discussion

### 2.1. Evolution of VpStyA1/VpStyA2B from Strain EPS

**Figure 1:**
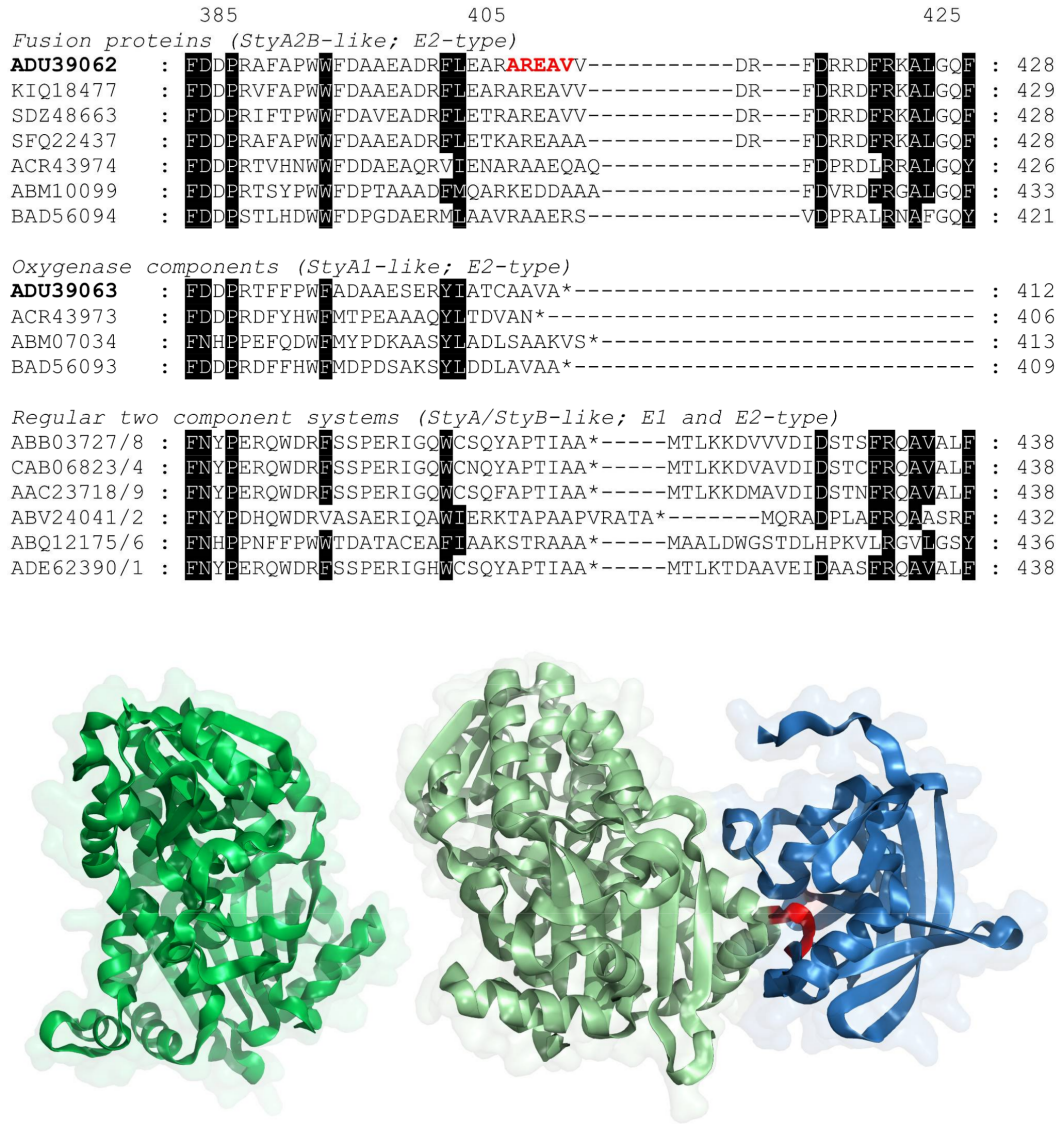
(Top) Sequence alignment of StyA2B-like proteins in comparison to StyA1 as well as functional StyA (left) and StyB (right) components. (Bottom) homology model of *Vp*StyA1 (darkgreen) *Vp*StyA2B (monooxygenase—lightgreen, reductase—blue). The proposed linker region is illustrated in red. Numbering on top is according to *Vp*StyA2B and the terminal amino acids are given at the end of lines. (ADU_*V. paradoxus* EPS; KIQ_*V. paradoxus* MEDvA23; SDZ_*Variovorax* sp. YR266; SFQ_*Variovorax* sp. OK605; ACR_*R. opacus* 1CP; ABM_*Paenarthrobacter aurescens* TC1; BAD_*Nocardia farcinica* IFM 10152; ABB_*Pseudomonas putida* SN1 (the A-component comprises the sequence GQWCSQY); CAB_*P. fluorescens* ST; AAC_*P. taiwanensis* VLB120; ABV_uncultured bacterium; ABQ_uncultured bacterium/MoxY; ADE_*Pseudomonas* sp. LQ26).

During an earlier study the putative genes encoding for the monooxygenase *Vp*StyA1/*Vp*StyA2B and the respective gene cluster of strain EPS were identified [21]. According to a phylogenetic analysis and to the surrounding genomic region the proteins were assigned as a two-component SMO related to the E2-prototype of *Rhodococcus opacus* 1CP [5,6,11]. Interestingly, the sequence similarity of *Vp*StyA1 and *Vp*StyA2B (74% identity over 404 amino acids) between both monooxygenase domains (A1 and A2) was much higher as among other StyA1/StyA2B systems. Furthermore, they form together a separate branch in a phylogenetic distance tree of monooxygenase components [21,23]. This phylogenetic study was now refined due to the release of more putative StyA1/StyA2B-sequences, especially from *Variovorax* species. This can help to identify the nature and position of the linker within the two domain proteins (StyA2B-like). The linker connects the monooxygenase (A2) and the FAD-reductase (B) domain (Figure 1). Respectively, the fusion event was discussed as functionally convergent event [21]. However, no activities of these putative E2-type SMOs originating of *Variovorax* species were reported until now.

Mining the databases for *styA2B*-like genes/proteins revealed that they occur mainly among Actinobacteria (e.g., *Amycolatopsis, Arthrobacter, Gordonia, Mycobacterium, Nocardia, Paenarthrobacter, Paeniglutamicibacter, Pseudarthrobacter, Sciscionella, Sinomonas, Streptomyces*, among others), but also among *Variovorax* (date of BLAST search: 19 September 2017; GenBank release 222). *Variovorax* belongs to the class of β-proteobacteria and not to Actinobacteria. There are no *styA2B*-like genes found in other none-Actinobacteria. However, there exist other *styA*/*styB-*genes encoding for protein components with a high sequence similarity to this E2-type SMOs among various genera. The closest homologues to *Variovorax* two domain proteins are found in *Delftia* although no natural fusion variant is present. 

Already with the first report of *Ro*StyA2B the linker region was discussed [5]. This can now be done with more emphasis on various sequences by means of sequence alignments as well as molecular modelling (Figure 1). A less conserved region/loop was identified as potential linker region. It is localized at the C-terminal site of the monooxygenase domain (A2) of StyA2B-like proteins. In case of *Vp*StyA2B it is located around region 408-AREAV-412. The alignment of StyA2B-, StyA1- and StyA-like amino acid sequences indicates that the last conserved amino acid among those proteins is at position 404 (*Vp*StyA2B) and in case of the none-fused proteins the following C-terminal sequences are variable. In case of the two domain proteins next to this region the domain of the NADH:FAD oxidoreductase (B-part) is localized. There the first conserved amino acid is present at position 417 (*Vp*StyA2B) with respect to related StyA2B- and StyB-like proteins. This region from 404 to 417 represents a flexible loop between two helices according to homology modelling. This is not surprising as there is no sequence-structure relation available for this part. However, this is in congruence to earlier made observations in which the N-terminal sequence of the FAD-reductase domain was investigated [9,20,21,24].

Adjacent to the monooxygenase domain (A2) and the proposed linker region the reductase domain (B) of the natural fusion proteins follows. Interestingly, the reductase sequence misses in all cases a few amino acids (range: 3 to 14) when compared to the StyB-like reductases of E1-type SMOs (Figure 1) [5,7]. The mentioned linker region was now chosen as a target for site-directed mutagenesis. In all cases the original sequence (AREAV) was replaced or even extended. The following mutants were successfully prepared and verified by sequencing: TIVVV, AAAAA, HHHHH, WYHHH, WYHHHHH, and GQWCSQY. In order to validate the made assumptions, the wildtype protein *Vp*StyA2B and the mutant proteins were produced and assayed. The chosen linker sequences of mutants base on the following rationale. Linker with A/V-rich sequences or H-rich were chosen to allow production of either more flexible or more rigid linker sequences, respectively [25,26]. Short H-rich sequences tend to form α–helical non-flexible structures [25,27]. This is well established for histidine-tagged proteins to allow a subsequent Ni-affinity purification. Often G/S-rich linker sequences are introduced between the H-tag and the target protein sequence to have more flexibility. The 408-GQWCSQY motif represents the C-terminal sequence of a StyA-protein originating from *Pseudomonas* (Figure 1; accession number: ABB03727) [28]. It was chosen to compare it to earlier made fusion proteins [19]. A more recent study reports on the generation and catalytic properties of an artificial E1-type SMO fusion protein comprising a long flexible linker [29]. This seemed to be promising for catalysis, but, does not resemble the naturally occurring fusion proteins.

### 2.2. Molecular Genetic Work and Enzyme Production

The cloning of both genes, *VpstyA1* and *VpstyA2B*, as well as the generation of mutants was successfully accomplished which was verified by sequencing the inserts of gene expression plasmids (See supporting information, Table S1) and by a simple indole based activity assay.

*E. coli* BL21 allows the formation of indole from tryptophan during growth on complex medium. Thus, the activity of styrene monooxygenases and related enzymes can be verified by indole transformation to yield indigo [4,5,6,10,11,20]. Indeed, all clones obtained [*E. coli* BL21 (DE3) pLysS derivatives harboring the wildtype or mutant genes in a pET-vector] produced indigo during cultivation even without being induced for overexpression of target proteins. Clones with highest indigo formation rate were selected, propagated and stored as glycerol stocks for later protein production and characterization.

In both cases (*Vp*StyA1 and *Vp*StyA2B), enzyme production was successfully achieved with a yield of 2 to 4 mg*_Vp_*_StyA2B_ and up to 9 mg*_Vp_*_StyA1_ protein per liter broth, respectively. This is in congruence to other studies [5,9,18]. In case of the mutants protein yields were significant lower.

### 2.3. Reductase Activity of VpStyA2B and VpStyA2B-Mutants

The fusion protein *Vp*StyA2B of strain EPS was supposed to be mainly a reductase of the complete monooxygenase system [6,7], and was for those reasons characterized in analogy to the enzyme *Ro*StyA2B of strain 1CP [5].

The protein *Vp*StyA2B was successfully produced (verified by SDS-PAGE, see supplemental material). The fraction obtained from Ni-chelate chromatography was slightly yellow, which is an indicator of a bound flavin. This was analyzed as described for other SMOs. FAD was determined by means of RP-HPLC as well as by spectroscopic methods and the use of authentic standards (see Supplemental Material). A FAD saturation of 4.9 to 20.8 mol% was calculated for the protein applied. This is in congruence to results obtained earlier for *Ro*StyA2B [6,18]. This fraction was immediately assayed for activity and then concentrated and stored at −20 °C in a suitable storage buffer until characterization. It used NADH as source of reducing equivalents in order to reduce flavins. NADH could not be replaced by NADPH. In case of the flavins FAD, FMN and riboflavin can be acceptors of reducing equivalents (Table 1). However, a clear preference was not determined with respect to catalytic efficiency which was for all employed flavins between 0.57 and 0.88 s^−1^ µM^−1^.

In contrast to the monooxygenase part (StyA2), promiscuity towards the flavin cosubstrate is in accordance with most characterized SMO reductases. So far, only one representative from strain 1CP (*Ro*StyB) is reported to be specific for FAD [9,23,24,30].

The reductase activity and catalytic efficiency of *Vp*StyA2B is up to 10-times higher than reported for the *Rhodococcus* enzyme *Ro*StyA2B [5]. Thus, differences at the amino acid level (57% identity) are reflected within the biochemical properties of StyA2B-like proteins. Still, the activity of the two domain protein is by an order of magnitude lower compared to most StyB-like reductases of other two-component systems [23,24,30]. Recently, it was reported that an N-terminal fusion for the StyB-like proteins drastically decreases the oxidoreductase activity [24]. In addition, an artificial fusion protein was constructed from two-component E1-type monooxygenase components of *Pseudomonas* [20]. Herein, the coupling (catalytic efficiency) was improved and the catalytic mechanism changed. It was also shown that the N-terminal region of the reductase influences the binding and affinity for the substrate [20,23]. Therefore, it is likely that the same is true for *Vp*StyA2B. However, it is likely that this effect of the fusion to StyA2 is different in dependence of the linker region and sequence in the *Variovorax* two domain enzyme.

*Vp*StyA2B was tested for inhibition or activation by a number of compounds used in other SMO studies (see supplementary material). The effects of the herein tested compounds are similar as observed for *Ro*StyA2B [5]. It can be concluded, that there is no metal dependency present. Moreover, in contrast to *Ro*StyA2B, the activity is lowered by addition of Fe-ions, while Fe^3+^ has a stronger effect. This is in accordance with inhibition studies on the reductase StyB from the two-component systems of *Pseudomonas taiwanensis* VLB120 and *Acinetobacter baylyi* ADP1 [23,30]. However, reducing agents as DTT and divalent ions as Ca^2+^ support the performance of *Vp*StyA2B. This was also observed for *Ro*StyA2B as well as *Ab*StyB. Interestingly, thioanisole decreases the reductase activity, which was not observed for SMO-reductases before.

In order to investigate the linker region of this two domain protein efforts to determine the respective region were accomplished on sequence level (see above) and served for the direction in a mutagenesis study. The six mutants of *Vp*StyA2B obtained were separately produced and purified as described above and characterized in analogy to the wildtype for their NADH:FAD oxidoreductase activities (Table 2). During expression also these clones produced significant amounts of indigo which indicated a functional expression of respective mutants.

All mutants were active and it was possible to determine specific activities (Table 2). Maximum specific activities were determined as described in materials and methods according to Michaelis-Menten. Excess of FAD did not change these values, but had to be assayed as the protein preparations might have different FAD-saturations as it was found for the wildtype two domain proteins [6,18]. The wildtype was most active with NADH and all the mutants showed a lower activity. However, the affinity for NADH seemed not to be altered as the *K*_m_ values were all in the same high range. That changed for FAD. The general activities were similar to those for NADH-variation and also the order from the most active to the worst representative was the same. But, the affinity for FAD was drastically different among the variants which was obvious from the *K*_m_ values determined and also reflected by the catalytic efficiencies (Table 2). The most efficient variant was AAAAA-mutant with respect to its oxidoreductase activity. Respectively, the catalytic efficiency of FAD-reduction was determined to 5.21 s^−1^ μM^−1^ which is about 8.1-times more efficient than the wildtype. This is due to the tighter FAD binding expressed by the 18.7-times lower *K*_m_ value for FAD. In addition, the mutants WYHHH and WYHHHHH showed an increased catalytic efficiency while the mutants HHHHH and GQWCSQY showed a decreased catalytic efficiency. Interestingly, mutant TIVVV was as efficient as the wildtype two domain protein but had a lowered specific activity.

Hence, the proposed and mutated linker region has a strong effect on the oxidoreductase activity. This might be due to a changed communication of domains (A2 and B) or due to altered FAD binding and turnover. This is in congruence to earlier studies which demonstrated that the N-terminal part of NAD(P)H:flavin oxidoreductases is important for the flavin binding and thus for catalysis [20,24]. Further, it indicates that the selected sequence region indeed can be the linker as it is functional relevant for the reductase domain.

### 2.4. Monooxygenase Activity of VpStyA1, VpStyA2B and VpStyA2B-Mutants

During the gene cloning and expression experiments the formation of indigo was observed and used to identify best protein producers. This was verified by a plate assay in order to get a view on the strains producing either a highly active SMO or a lot of protein (Table 3). The wildtype proteins *Vp*StyA1 and *Vp*StyA2B and especially the AAAAA-mutant produced most indigo. The acceptance of indole as a substrate and the formation of indigo fits to very recent findings that related E2-type two-component monooxygenases were assigned to indole degradation and act basically as indole monooxygenases [13,14].

The wildtype monooxygenase components *Vp*StyA1 and *Vp*StyA2B show a comparable epoxidase activity as other SMOs (range 0.02 to 2.1 U mg^−1^) (Table 3) [5,6,7,8,9,10,30]. *Vp*StyA1 is about 2.9-times and 2.1-times more active than *Vp*StyA2B and the *Rhodococcus* counterpart *Ro*StyA1, respectively. And it was confirmed that *Vp*StyA1 represents the major monooxygenase of the system *Vp*StyA1/*Vp*StyA2B as it was found for the prototype *Ro*StyA1/*Ro*StyA2B [6]. This was expected but also indicates that the high sequence similarity of the *Variovorax* monooxygenases does not change the catalytic properties to a large extent. However, *Vp*StyA1/*Vp*StyA2B does not reach the high epoxidase activity of StyA of strain VLB120 (2.1 U mg^−1^) [30].

It is interesting that *Vp*StyA2B is about 8.4-times more active on styrene than *Ro*StyA2B [5,6]. The activity of the latter could be boosted by additional FAD-reductase, but, not for *Vp*StyA2B. Another difference between both StyA2B-proteins is the FAD-reductive power which is about 4.3-times higher in the case of *Vp*StyA2B with a *k*_cat_ of 22.3 s^−1^ vs. that of *Ro*StyA2B of 5.2 s^−1^. This higher oxidoreductase efficiency of *Vp*StyA2B might lead to the higher epoxidase activity, even, without an additional FAD-reductase. Hence, the two domain protein *Vp*StyA2B is a better biocatalyst as the prototype *Ro*StyA2B. 

The mutants obtained were in analogy to the wildtype enzymes characterized for their capability to convert styrene into styrene oxide. The wildtype *Vp*StyA2B showed an activity of 159 mU mg^−1^. The *Vp*StyA2B-variants were prepared similarly and assayed immediately in order to allow a direct comparison. The following relative styrene epoxidase activities were determined: 84.9% for TIVVV, 163.5% for AAAAA and less than 2% in case of the other variants (HHHHH, WYHHH, WYHHHHH, and GQWCSQY). This clearly shows the proposed linker region has significant effects on the epoxidase activity of StyA2B-proteins.

From the nature of this proposed linker sequence it can be reasoned that smaller hydrophobic residues yield higher activities compared to larger or more polar residues. This is indeed true for the FAD-reductase as well as for the corresponding styrene epoxidase activity. And again the mutant 408-AAAAA showed a very promising catalytic behavior which was better than the wildtype in terms of activity and efficiency (Table 2 and Table 3). This means the electron transfer from NADH towards FAD which yields reduced FAD and allows oxygen activation for catalysis was more efficient in case of mutant protein AAAAA (an electron transfer yield of 3). In terms of epoxidase activity it was followed by the TIVVV-mutant (an electron transfer yield of 4.4 at a lower epoxidation activity) which was even more active with surplus of reduced FAD supplied by an additional FAD-reductase (Table 3; extra *Ro*StyBart). It achieved almost the same epoxidase activity as *Vp*StyA1. This might allow to draw some conclusions as it might be possible to generate more efficient and catalytic active self-sufficient two domain proteins; here SMO-like monooxygenases. But, as the mutagenesis of the C-terminal part of the StyA2-domain improved catalytic properties it might be possible to alter StyA1- or StyA-like proteins at their C-terminal site in order to improve their catalytic properties. Furthermore, the generation of artificial fusion proteins seems promising [20,29]. However, structural investigations are necessary in order to elucidate the nature of substrate and cofactor binding.

Various flavin-dependent monooxygenases accept sulfide-like compounds as substrates [4,5,6,7,8,9,10,11]. The latter can be converted to sulfoxides or sulfones in dependence of the employed biocatalyst [1,2,3]. Therefore, the specific activity of *Vp*StyA1/*Vp*StyA2B on a model substrate, here benzyl methyl sulfide (BMS), was determined. Specific activities were determined as described above for styrene in order to allow a comparison (Table 2). Maximum specific activity on BMS was determined to 3.113 U mg^−1^ *Vp*StyA1 and 1.616 U mg^−1^ *Vp*StyA2B, respectively. This is about 25 to 26-times faster as the specific activity on styrene. Hence, the two-component monooxygenase of strain EPS has a preference for this sulfide. Also this high sulfoxidase activity is comparable to other SMOs [8,10], but the high selectivity yielding almost pure (*S*)-enantiomers is somewhat outstanding.

### 2.5. Biotransformation of Sulfides

For the biotransformation experiments, another fresh batch the enzyme (*Vp*StyA1 and *VpS*tyA2B) was produced and partially purified. The enriched protein fractions obtained were sufficient to study substrate range and conversion. Sulfoxides were chosen as target compounds since *Vp*StyA1/*VpS*tyA2B showed an enhanced sulfoxidase activity. Respective sulfides were used as substrates (Table 4). Both components converted the sulfides into corresponding sulfoxides. A further oxidation to sulfones was not observed. And BMS was the best substrate. *Vp*StyA1 produces almost exclusively the (*S*)-sulfoxides and good conversions of up to 95% were achieved. *Vp*StyA2B also preferably produces the (*S*)-enantiomer but at a lower enantiomeric excess. The overall conversions were much lower in case of the two domain protein. This is in congruence to the kinetic investigations and indicates that *Vp*StyA1 is the main monooxygenase of the two-component system.

Earlier reports on SMO-based sulfoxidations showed often a high activity but low enantioselectivity [1,2,3,4,5,6,7,8,9,10]. This is now improved by means of *Vp*StyA1, which shows excellent conversions of sulfides in short reaction times while also reaching a high ee-value of the respective products (Table 4). It allows access to the (*S*)-enantiomers. It would be also interesting to get an enzyme producing the (*R*)-enantiomers with a high ee. A monooxygenase from metagenome allows one to produce (*R*)-enantiomers, but at rather low ee-values (60–92%) and after much longer reaction times [10].

## 3. Materials and Methods 

### 3.1. Synthesis of Sulfoxides

Commercial sulfides served as base to produce sulfoxides which were applied as standards for analytical methods. The chemical sulfoxidation was achieved according to the protocol published earlier [31] as it provided high yields of desired products. First a solution of 7 mmol sulfide in 100 mL methanol and 20 mL water plus 10 mL titanium (III) chloride (16% aqueous solution) was prepared. Then dropwise a hydrogen peroxide solution (3.2 mL 30% aqueous in 15 mL methanol) was added while constantly stirring at ambient temperature (about 20 °C). Latest after 25 min the substrate was completely converted and the reaction had been stopped by adding 50 mL water. Sulfoxides formed were extracted by chloroform (three times) and subsequently dried over anhydrous magnesium sulfate. Chloroform was further removed under reduced pressure. Success of reactions and purity of products was determined as described elsewhere [32].

### 3.2. Nucleotides, Sequence Analysis and Molecular Modelling

Sequence analyses based on a previously performed investigation [5]. Thus accession numbers of the E2-type SMO components originating of *Variovorax paradoxus* EPS were already available: ADU39063 (*Vp*StyA1) and ADU39062 (*Vp*StyA2B). These were used for a BLASTP search in order to identify related or even homologous proteins. This allowed to generate an amino acid sequence alignment in analogy to earlier reports [5,21]. This served as a template for the subsequent calculation of a dendrogram and the homology modelling. As templates for homology modelling the two available structures (PDB StyA: 3IHM, and PDB PheA2: 1RZ0) were employed [19,33]. The C-terminal StyA2-part was linked to the N-terminal StyB-part by a random loop which gets obvious from Scheme 1 and Figure 1, respectively. The following tools were used: MEGA7 for the alignment, Modeller program version 9.19 for the comparative homology modelling as well as GenDoc and PyMol V1.1r1 for alignment and 3D-structure visualization [9,34,35,36].

### 3.3. Bacterial Strains and Cultivation

*Escherichia coli* strains DH5α and BL21 (DE3) pLysS were cultivated for cloning and expression as described elsewhere [5,6,9,37]. A list of genes, primers and plasmids for this work is presented in the Supplemental Information (Table S1). Indigo formation was observed during the cultivation and gene expression experiments. A plate screening in order to monitor indigo formation was set up as follows. The expression clones were transferred on a M9 mineral media agar-plate in a predefined grid, followed by a 22 h incubation at 30 °C [38]. The solid media was supplemented with antibiotics as described previously and 0.5 mM isopropyl-β-d-thiogalactopyranoside for induction of protein expression [5,6,9,37]. Indigo formation was started by spraying the plates with an indole-substrate solution (5 Mm Tris-HCl, pH 7.5, 50 mM indole, 20% DMSO). The plates were then transferred onto a white light-table in a darkened box equipped with a camera. Pictures of the plates were taken in a 30 s interval. The color formation of each colony was monitored and normalized upon colony size to get a time-resolved intensity profile for each clone.

### 3.4. Molecular Genetic Work

The DNA sequences of *VpstyA1* (Accession number: MF781076) and *VpstyA2B* (MF781075) were optimized for the codon usage and GC content of *Acinetobacter baylyi* ADP1 as described previously [9,39]. The genes were purchased in a pEX-A vector system from Eurofins MWG (Ebersberg, Germany) with 5′-NdeI and 3′-NotI restriction sites allowing for subcloning into pET16bP [9]. Site-directed mutagenesis of the linker area was done by using the GeneMorph II EZClone Domain Mutagenesis Kit (Agilent Technologies, Ratingen, Germany). A megaprimer was generated by amplifying the target region with a primer that contains the desired mutation. This megaprimer was annealed to the parental plasmid and extended in the EZClone reaction. Afterwards, the parental DNA was DpnI digested and the remaining pET16bP construct harboring the mutation was transformed into *E. coli* BL21 cells for gene expression. Successful mutation of the target genes was proven by sequencing of the plasmid using the pET16-check-fw/pET16-check-rev primer [40].

### 3.5. Protein Purification and Quantification

Recombinant protein production and purification was done as described previously [9,24]. Protein concentration was determined by the Bradford method employing bovine serum albumin as a standard [41]. The purity of protein preparations was controlled by SDS-PAGE as described earlier for those types of proteins [5,6]. Further, the FAD content of proteins was determined by the protocol employed for *Ro*StyA1 and *Ro*StyA2B [6]. Therefore, authentic standards for FMN and FAD had been determined by RP-HPLC connected to a diode array detector for UV/Vis range.

### 3.6. Enzyme Assays and Product Analysis

Flavin oxidoreductase activity was determined spectrophotometrically (Cary 50, Varian, Agilent Technologies, Ratingen, Germany) by quantifying NAD(P)H consumption at 340 nm (ε_340_ nm = 6.22 mM^−1^ cm^−1^). One unit of enzyme activity is defined as the amount required to oxidize 1 µmol of NAD(P)H per min. All measurements were carried out in triplicate. The standard assay (1 mL) consisted of 20 mM Tris-HCl, pH 7.5, 68 µM flavin co-substrate (FAD, FMN or riboflavin) and 177 µM NADH. After incubating the mixture for 10 min at 30 °C, the reaction was started by adding an appropriate amount of enzyme. For estimating steady-state specific activities, initial reaction rates were determined using 4.1 to 90.2 µM FAD and 7.9 to 164 µM NADH. Specific activities were obtained by nonlinear regression analysis applying KaleidaGraph 4.5 (Synergy Software, Reading, PA, USA), assuming Michaelis-Menten kinetics.

Monooxygenase activity assays and standard HPLC analytics were performed as described previously [5,9]. Reduced FAD was supplied by an additional reductase *Ro*StyBart [24] or by chemical reduction with 10 mM BNAH, respectively. The 2 h biotransformations were done according to the standard monooxygenase assay by adding the respective sulfides instead of styrene as substrate. Enantiomeric excess of obtained products were analyzed as described previously for epoxides [5] and sulfoxides [32].

## 4. Conclusions

The two-component monooxygeanse *Vp*StyA1/*Vp*StyA2B originating of *Variovorax paradoxus* EPS was herein described as the first proteobacterial E2-type representative. It also comprises a two domain protein as an NADH:FAD-oxidoreductase (*Vp*StyA2B). Both proteins were successfully produced by recombinant gene expression. The enzyme converts styrene in an enantioselective manner as well as several aryl alkyl sulfides. However, it was by far more active (3.3 to 22-times) on these sulfides as on styrene. Therefore, this two-component enzyme can be proposed as an enantioselective sulfoxidase. Furthermore, the potential linker region of the two domain StyA2B-like protein was identified and mutated. It was demonstrated that this linker region has effects on the reductase (B) as well as monooxygenase (A2) domain. Structural and kinetic studies may reveal the effect of the linker on the individual domains of *Vp*StyA2B and related monooxygenase. Thus, this monooxygenase of strain EPS is a suitable candidate for structural investigations as well as to develop an enantioselective catalyst for sulfoxidation reactions.

## Figures and Tables

**Table 1:** NADH:flavin oxidoreductase activity of *Vp*StyA2B (MW = 66.32 kDa which was calculated from the amino acid sequence including the N-terminal tag).

Donor ^1^/Acceptor (µM) ^2^	*K*_m_ (μM)	V_max_ (U mg^−1^)	*k*_cat_ (s^−1^)	*k*_cat_ *K*_m_^−1^ (s^−1^ μM^−1^)
NADH (7.9–164)/FAD (70)	24.0 ± 4.0	16.2 ± 0.8	17.9 ± 0.9	0.72
NADH (164)/FAD (6.3–78.8)	33.6 ± 4.0	20.2 ± 1.0	22.3 ± 1.1	0.64
NADH (164)/FMN (4.1–90.2)	45.9 ± 6.8	26.0 ± 1.8	28.7 ± 1.9	0.57
NADH (164 µM)/Riboflavin (6.3–88.2)	37.7 ± 7.2	31.3 ± 2.7	34.6 ± 2.9	0.88

^1^ NADPH (230 µM in presence of 70 µM FAD) did not serve as an electron donor. ^2^ Either electron donor or acceptor was present in excess and the data obtained of triplicates were analyzed assuming Michaelis-Menten kinetics.

**Table 2:** NADH:flavin oxidoreductase activity of *Vp*StyA2B-mutants and -wildtype.

*Vp*StyA2B-Variant	Donor NADH ^1^			Acceptor FAD ^1^		
	*K*_m_ (μM)	V_max_ (U mg^−1^)	*k*_cat_ *K*_m_^−1^ (s^−1^ μM^−1^)	*K*_m_ (μM)	V_max_ (U mg^−1^)	*k*_cat_ *K*_m_^−1^ (s^−1^ μM^−1^)
wildtype *Vp*StyA2B	24.0	16.2	0.72	33.6	20.2	0.64
408-TIVVV	37.7	3.3	0.09	5.1	3.1	0.65
408-AAAAA	28.1	8.9	0.34	1.8	8.8	5.21
408-HHHHH	44.2	2.5	0.06	14.5	3.1	0.23
408-WYHHH	20.1	7	0.37	3.2	6.9	2.30
408-WYHHHHH	40.6	13.8	0.36	7.4	13.2	1.90
408-GQWCSQY	47.6	3.8	0.09	14.5	3.7	0.27

^1^ In order to determine kinetic properties concentrations of NADH and FAD were chosen as for the wildtype *Vp*StyA2B given in Table 1. Triplicates were used to get the values and standard deviations were in all cases less than 15% and comparable to Table 1.

**Table 3:** Epoxidase activity of *Vp*StyA2B-mutants and -wildtype enzymes.

Substrate	Styrene			Indole
SMO ^1^	V_max_ (mU mg^−1^)	ETY ^2^ (%)	V_max_ (mU mg^−1^) + Extra *Ro*StyBart ^3^	Plate Screening ^4^
wildtype *Vp*StyA1	n.a.	n.a.	460	+++
wildtype *Vp*StyA2B	159	1	140	++
408-TIVVV	135	4.4	327	+
408-AAAAA	260	3	260	+++
408-HHHHH	<1	<1	4	+
408-WYHHH	1.7	<1	7.3	+
408-WYHHHHH	<1	<1	1.6	+
408-GQWCSQY	<1	<1	3.1	+

^1^ Mutants are made of *Vp*StyA2B as described in methods and were in analogy to the wildtype investigated. ^2^ The electron transfer yield (ETY) was calculated from the NADH-consumption vs. epoxidation rate. ^3^ In case of StyA1 an additional NADH:FAD oxidoreductase was needed (here *Ro*StyBart) which could also assist StyA2B-like proteins [6,24]. ^4^ The plate screening was performed with clones expressing respective genes on an agar plate and the indigo formation (+ little, ++ normal, and +++ significant indigo formed) was followed online by a camera. n.a. = no activity measureable.

**Table 4:** 2 h Biotransformation of sulfides and styrene. Conversions of 2 mM substrate were analyzed.

Substrate	Product	*Vp*StyA1 Conversion (%)	ee (%)	*Vp*StyA2B Conversion (%)	ee (%)
PMS	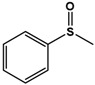	43.6 ± 1.9	98 (*S*)	6.3 ± 0.3	64 (*S*)
4F-PMS	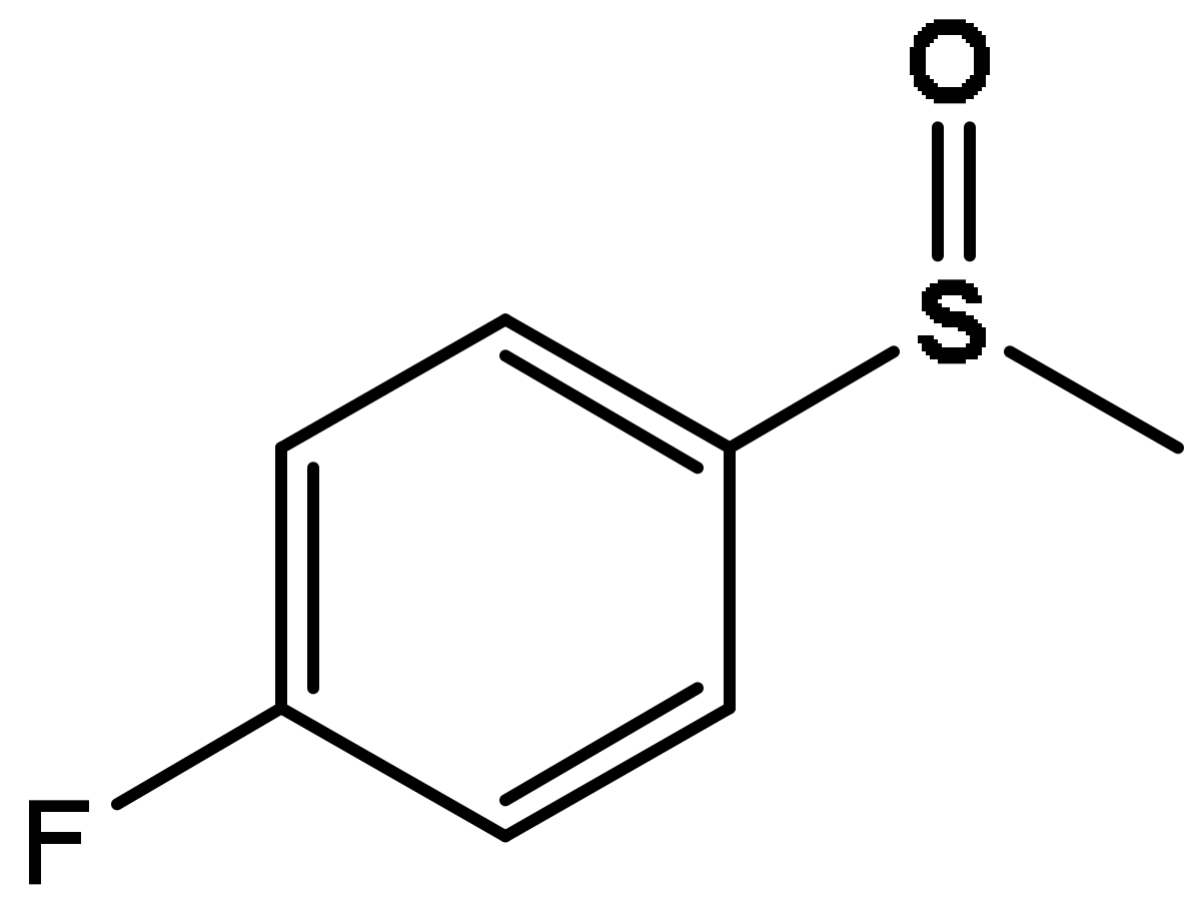	59.8 ± 0.4	99 (*S*)	4.3 ± 0.7	84 (*S*)
4Cl-PMS	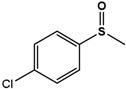	38.5 ± 1.7	99 (*S*)	4.4 ± 0.4	96 (*S*)
4Br-PMS	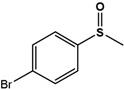	32.4 ± 4.7	99 (*S*)	6 ± 0.7	96 (*S*)
BMS	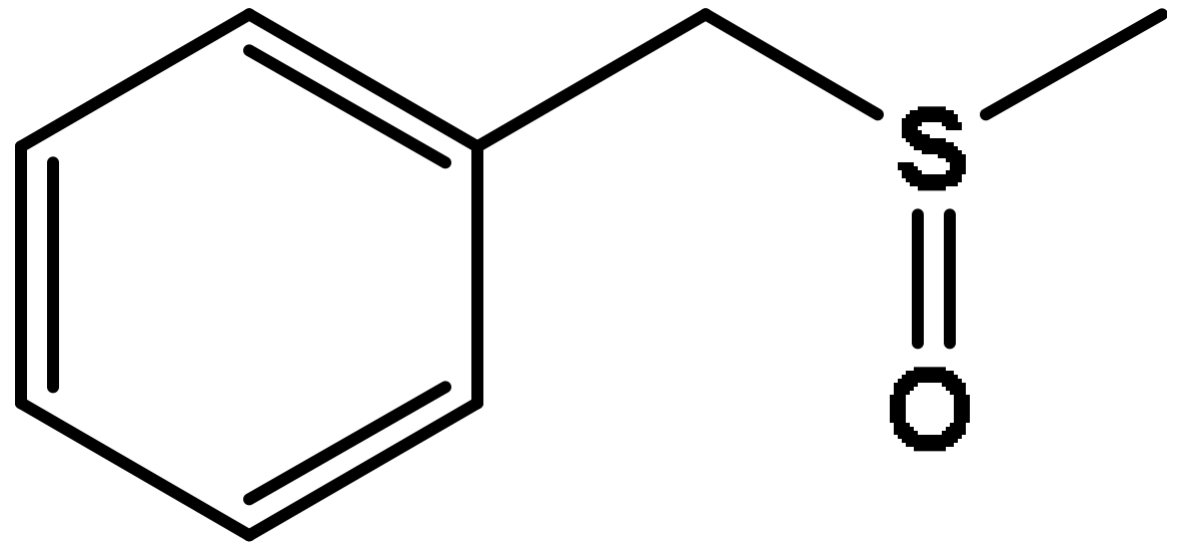	95 ± 0.5	97 (*S*)	13 ± 0.9	n.d. (*S*)
Styrene	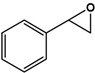	9.9 ± 0.4	98.2 (*S*)	0.5 ± 0.1	45 (*S*)

Phenyl methyl sulfide (PMS; thioanisole), 4-fluoro phenyl methyl sulfide (4F-PMS), 4-chloro phenyl methyl sulfide (4Cl-PMS), 4-bromo phenyl methyl sulfide (4Br-PMS), benzyl methyl sulfide (BMS); n.d. = not detectable.

## Supplementary Materials

Supplementary materials are available online. Figure S1: Sensitivity of *Vp*StyA2B towards putative inhibitors determined by applying the NADH:FAD oxidoreductase assay, Figure S2: Flavin determination of denatured *Vp*StyA2B, Figure S3: SDS-PAGE analysis of protein preparations: *Vp*StyA1 and *Vp*StyA2B, Table S1: Strains, plasmids and primers used in this study.
