# Supplementary material for: VpStyA1/VpStyA2B of Variovorax paradoxus EPS: An Aryl Alkyl Sulfoxidase Rather than a Styrene Epoxidizing Monooxygenase
Source: Molecules. 2018 Apr 2;23(4):809. doi: 10.3390/molecules23040809 (PMC6017014; doi:10.3390/molecules23040809)
Supplement: Supplementary file 1 [file molecules-23-00809-s001.pdf]

*Vp*StyA1/*Vp*StyA2B of *Variovorax paradoxus* EPS: rather an aryl alkyl sulfoxidase than a styrene epoxidizing monooxygenase

Dirk Tischler*[a], Ringo Schwabe[a], Lucas Siegel[a], Kristin Joffroy[a], Stefan R. Kaschabek[a], Anika Scholtissek[a] and Thomas Heine[a]

[a] Dr. D. Tischler *(corresponding author), R. Schwabe, L. Siegel, K. Joffroy, Dr. S. R. Kaschabek, A. Scholtissek, T. Heine
Institute of Biosciences, Environmental Microbiology
TU Bergakdemie Freiberg
Leipziger Str. 29, 09599 Freiberg, Germany
E-mail: dirk-tischler@email.de

Supporting information for this article is given via a link at the end of the document.

SUPPLEMENTARY MATERIAL

Table S1. Strains, plasmids and primers used in this study.

Strain, plasmid, or primer	Relevant characteristic(s)	Source / Reference
E. coli DH5α	F^- φ80d*lacZ* M15 (*lacZYA-argF*)U169 *endA1 recA*1 *hsdR*17(rK$^-$, mK$^+$) *supE*44 λ^- *thi*-1 *gyrA*96 *relA*1	Gibco-BRL
E. coli BL21(DE3) (pLysS)	*hsdS gal* (λcI*ts*857 *ind*1 Sam7 *nin*5 *lac*UV5-T7 gene 1), pLysS (Cm^R)	Stratagene
pEX_A_VpstyA1	*VpstyA1* of *V. paradoxus* EPS (~1.25 kb NdeI/KpnI-fragment) cloned in pEX vector with additional multiple cloning site, (Amp^r)	This study Eurofins MWG
pEX_A_VpstyA2B	*VpstyA2B* of *V. paradoxus* EPS (~1.75 kb NdeI/KpnI-fragment) cloned in pEX vector with additional multiple cloning site, (Amp^r)	This study Eurofins MWG
pET16bP	pET16b (Novagen) with additional multi-cloning site, allows expression of recombinant proteins with N-terminal 10x His-tag	Wehmeier (pers. comm)
pSVpstyA1_P01	*VpstyA1* of *V. paradoxus* EPS (~1.25 kb NdeI/KpnI-fragment) cloned in pET16bp	This study
pSVpstyA2B_P01	*VpstyA2B* of *V. paradoxus* EPS (~1.75 kb NdeI/KpnI-fragment) cloned in pET16bp	This study
pSVpAAAAA_P01	*VpstyA2_408-AAAAA_B* gene in pET16bP (plasmid generated by site-directed mutagenesis using a fw_AAAAA/pET16-check-rev PCR-fragment as megaprimer)	This study
pSVpHHHHH_P01	*VpstyA2_408-HHHHH_B* gene in pET16bP (plasmid generated by site-directed mutagenesis using a fw_HHHHH/pET16-check-rev PCR-fragment as megaprimer)	This study
pSVpWYHHH_P01	*VpstyA2_408-WYHHH_B* gene in pET16bP (plasmid generated by site-directed mutagenesis using a fw_WYHHH/pET16-check-rev PCR-fragment as megaprimer)	This study
pSVpGQWCSQY_P01	*VpstyA2_408-GQWCSQY_B* gene in pET16bP (plasmid generated by site-directed mutagenesis using a fw_GQWCSQY /pET16-check-rev PCR-fragment as megaprimer)	This study
pSVpWYHHHHH_P01	*VpstyA2_408-WYHHHHH_B* gene in pET16bP (plasmid generated by site-directed mutagenesis using a fw_WYHHHHH/pET16-check-rev PCR-fragment as megaprimer)	This study
pET16-check-fw	CATCACAGCAGCGGCCATATCGAAG	[1]
pET16-check-rev	CAGCTTCCTTTCGGGCTTTGTTAG	[1]
fw_AAAAA	TTCCTGGAAGCACGTGCGGCCGCGGCCGCGGTTGACCGCTTTGATC	This study
fw_HHHHH	TTCCTGGAAGCACGTCATCACCATCACCATGTTGACCGCTTTGATC	This study
fw_WYHHH	TTCCTGGAAGCACGTTGGTATCACCACCACGTTGACCGCTTTGATC	This study
fw_GQWCSQY	TTCCTGGAAGCACGTGGCCAGTGGTGCAGCCAGTATGTTGACCGCTTTGATC	This study
fw_WYHHHHH	TTCCTGGAAGCACGTTGGTATCACCACCACCACCACGTTGACCGCTTTGATC	This study
fw_TIVVV	TTCCTGGAAGCACGTACCATAGTGGTGGTGGTTGACCGCTTTGATC	This study

Primer sequence direction is 5’→3’

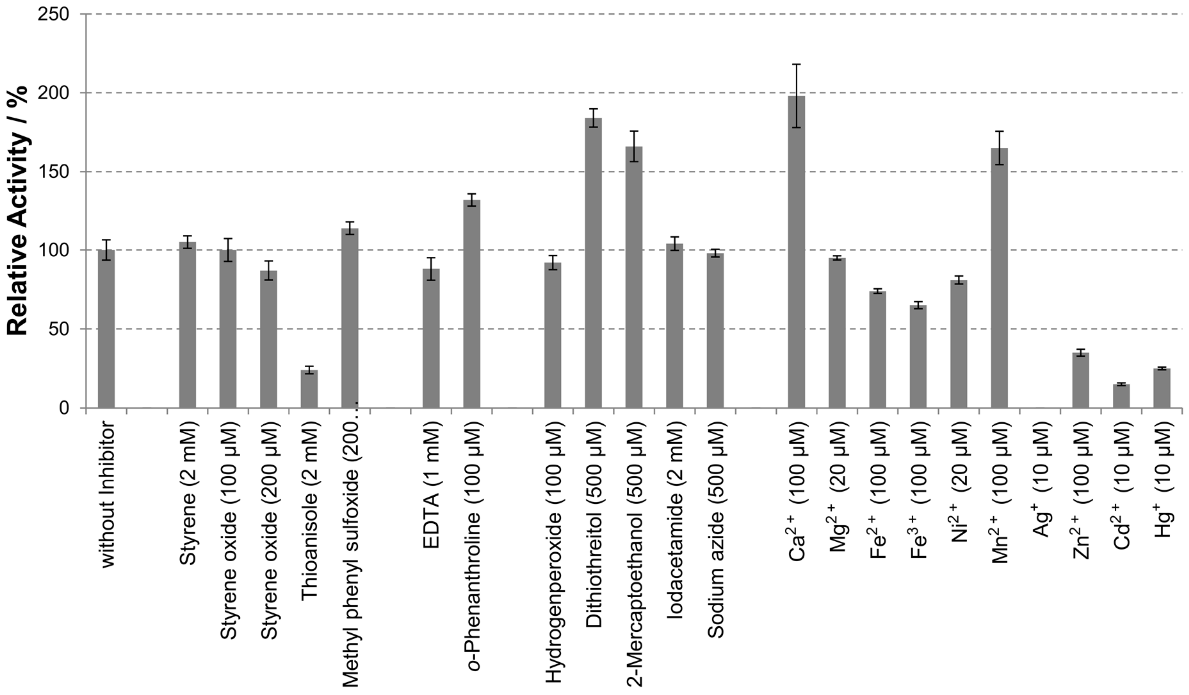

Figure S1. Sensitivity of *Vp*StyA2B towards putative Inhibitors determined by applying the NADH:FAD oxidoreductase assay.

A) Sample from protein preparation; *Vp*StyA2B

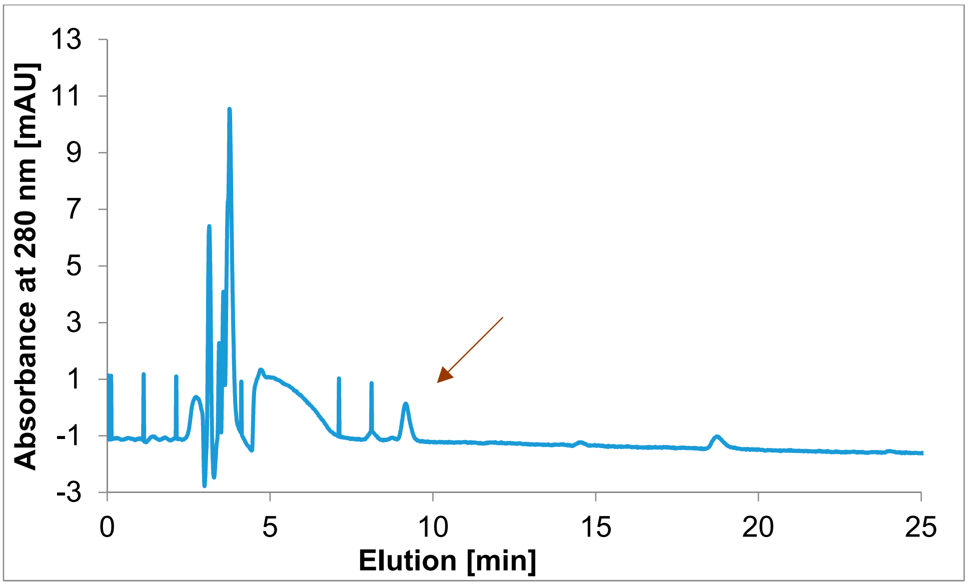

B) FAD standard

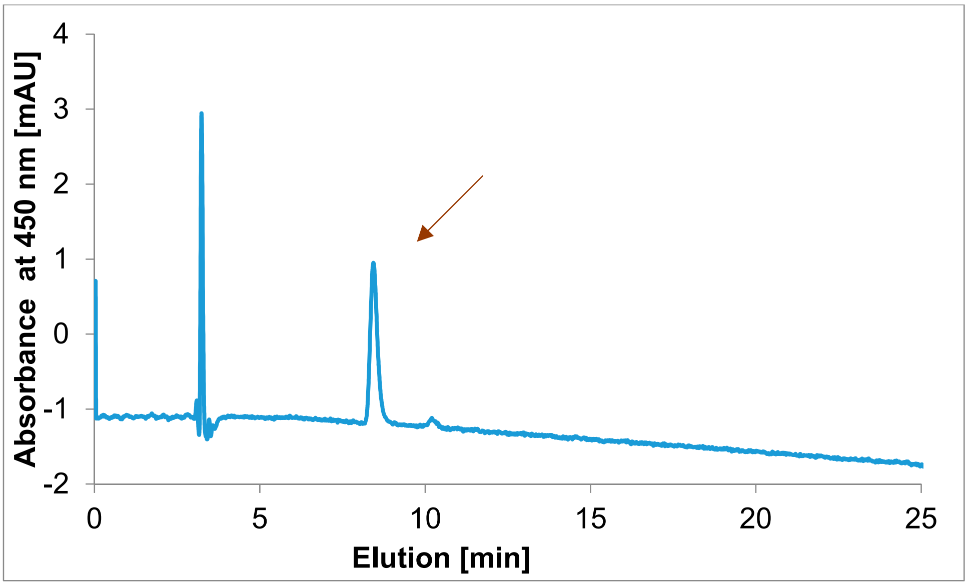

Figure S2. Flavin determination of denatured *Vp*StyA2B

The result obtained from protein preparation (A) showed a peak at the same elution volume as the standard of FAD shown in (B). Riboflavin and FMN had been determined as well, but only as standards and not from protein preparation.

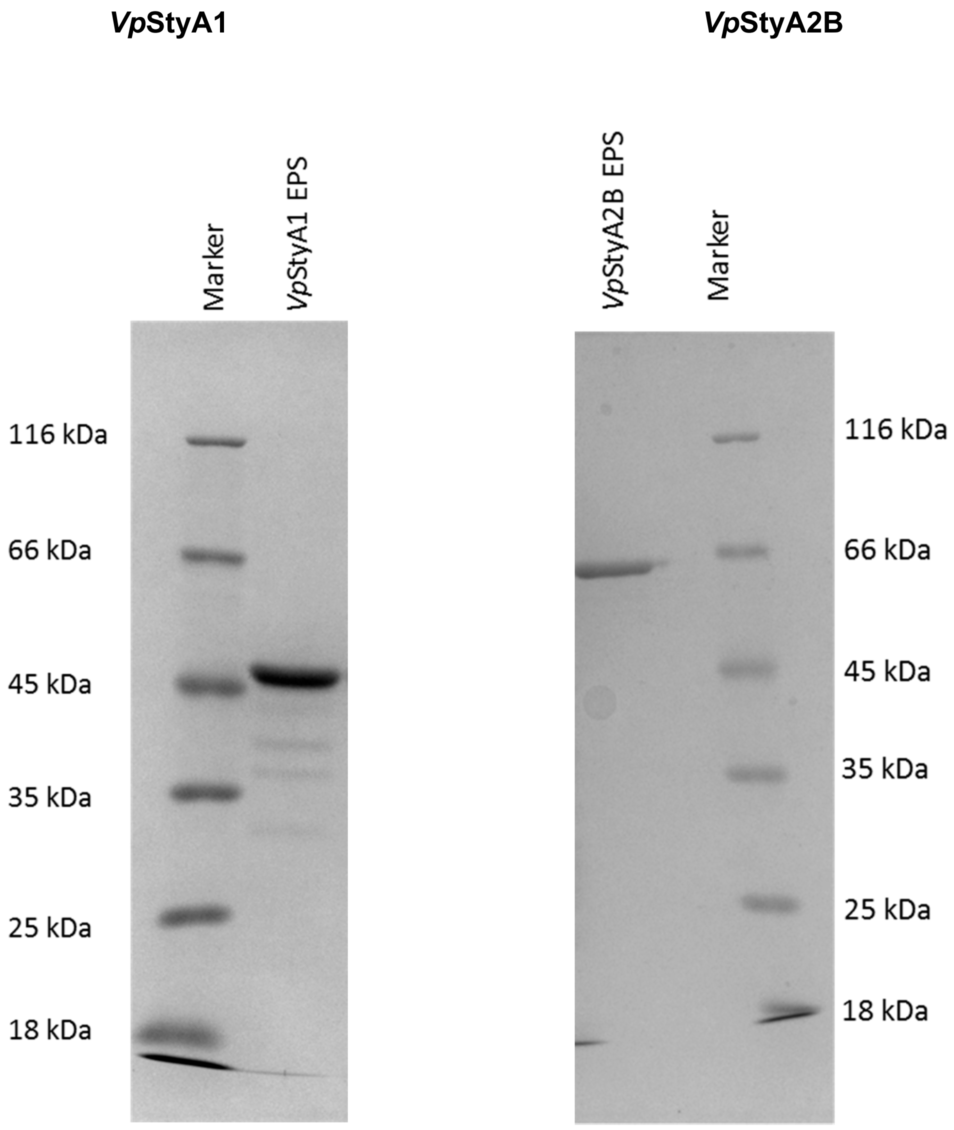

Figure S3. SDS-PAGE analysis of protein preparations: *Vp*StyA1 and *Vp*StyA2B

Reference

[1] J. Qi, M. Schlömann, D. Tischler, *J. Mol. Catal., B Enzym*. **2016**, *130*, 9–17.
